# Cell Transfer Therapy for Cancer: Past, Present, and Future

**DOI:** 10.1155/2014/525913

**Published:** 2014-01-09

**Authors:** Xiaoling Qian, Xian Wang, Hongchuan Jin

**Affiliations:** Department of Medical Oncology, Institute of Clinical Science, Sir Run Run Shaw Hospital, School of Medicine, Zhejiang University, Hangzhou, China

## Abstract

Cell transfer therapy for cancer has made a rapid progress recently and the immunotherapy has been recognized as the fourth anticancer modality after operation, chemotherapy, and radiotherapy. Lymphocytes used for cell transfer therapy include dendritic cells, natural killer (NK) cells, and T lymphocytes such as tumor-infiltrating lymphocytes (TILs) and cytotoxic T lymphocytes (CTLs). In vitro activated or engineered immune cells can traffic to cancer tissues to elicit persistent antitumor immune response which is very important especially after immunosuppressive treatments such as chemotherapy. In this review, we overviewed recent advances in the exploration of dendritic cells, NK cells, and T cells for the treatment of human cancer cells.

## 1. Introduction

Inspired by the observation of complete tumor regression in a male patient with recurrent sarcoma after a postoperative infection of erysipelas, Coley treated advanced sarcoma patients with mixed toxins of streptococcus erysipelas and bacillus prodigiosus in 1891 [1, 2], thus starting the history of the immunotherapy for human cancers. Unfortunately, limited progress has been achieved since then. Recently, the great successes in adoptive cell transfer therapy (ACT) and the development of anti-cancer antibodies such as Ipilimumab rekindled the interest of the scientific community in the anticancer immunotherapy and cytokine treatments. Now, the immunotherapy has been recognized as the fourth anticancer modality after operation, chemotherapy, and radiotherapy.

There are two types of immunotherapy for cancer, active immunotherapy and passive immunotherapy. The active immunotherapy mainly refers to vaccines, immune adjuvants, and cytokines, while the passive therapy consists of immune modulating antibody-based therapy and adoptive immunotherapy. Active immunotherapies can activate endogenous immune system and passive immunotherapies provide or strengthen immune reaction in cancer patients by infusing antibodies or effector cells produced in vitro. Among the active immunotherapy, cytokines including interleukin-2 (IL-2), interleukin-12 (IL-12), granulocyte-macrophage colony-stimulating factor (GM-CSF), tumor necrosis factor (TNF)-*α*, and interferons (IFNs) are secreted by immune cells and play pivotal roles in the active immunotherapy. IL-2 is an important growth factor for lymphocytes. It has been proved by FDA to treat advanced melanoma and renal carcinoma. However, the serious systemic toxicities of high-dose IL-2 restrict its wide application [3].

Depending on the different immunocytes transferred, cell transfer therapy (CTT) includes active immunotherapy and passive immunotherapy or ACT. Adoptive cell transfer therapy uses patient-specific autologous or HLA-matched allogeneic lymphocytes activated in vitro with growth factors while dendritic cells were frequently used in active cell transfer therapy to elicit antitumor immune response. The immunocytes used in ACT can be divided into at least two types. The first type includes lymphokine-activated killer (LAK) cells, CIK (cytokine-induced killer) cells, and NK cells, all of which can mediate cancer regression with non MHC restriction. Another type of effector cells refers to the tumor-infiltrating lymphocytes (TILs) and cytotoxic T lymphocytes (CTL), both of which recognize specific tumor antigens presented by MHC molecules.

## 2. DC-Based Cell Transfer 

After the identification of dendritic cells (DC) as the most efficient antigen presenting cells (APC) in vivo [4], DC-based vaccines have received more interests. Dendritic cells possess special properties of mediating crosstalk between innate and adaptive immune responses, and mature DCs can stimulate activation of autologous tumor-specific CD8+ T cells to reduce the tumor mass [5]. DC can be isolated form peripheral blood mononuclear cells (PBMC), expanded in vitro and challenged with a wide variety of cancer-specific antigens.

Sipuleucel-T (Provenge) is the first therapeutic cancer vaccine approved by the US Food and Drug Administration (FDA) for the treatment of castration-resistant minimally symptomatic prostate cancer in April 2010. Sipuleucel-T is a DC-based vaccine which is expanded ex vivo with a fusion protein (PA2024) comprising of granulocyte-macrophage colony-stimulating factor (GM-CSF) and prostatic acid phosphatase [6]. Three important phase III trials manifested benefits to improve overall survival in advanced prostate cancer [7–9]. In a meta-analysis of randomized clinical trials with a total of 737 patients with metastatic castrate-resistant prostate cancer (mCRPC), the overall survival of patients who received sipuleucel-T in comparison to the placebo treatment was significantly longer with a hazard ratio (HR) for overall survival of 0.73 (95% CI: 0.61–0.88; *P* = 0.001). However, time for disease progression was not prolonged (HR = 0.89, 95% CI: 0.75–1.05; *P* = 0.18). Remarkably, no serious side effect was reported. Compared to the control group, the pooled relative risks (RR) of all adverse events (RR = 1.03, 95% CI: 1.00–1.05; *P* = 0.06), grade 3 to 5 adverse events (RR = 0.98, 95% CI: 0.79–1.22; *P* = 0.86), and cerebrovascular events (RR = 1.93, 95% CI: 0.73–5.09; *P* = 0.18) were not significantly higher for men treated with sipuleucel-T. There are more reports from phase II/III trials showing promising clinical outcomes of DC-based vaccines and the outcomes are related with the vaccine-induced expansion of tumor-specific effector T cells [10, 11].

Immature DCs not only function poorly in antigen presentation but also induce immune tolerance [5]. Therefore, it is crucial to promote maturation and differentiation of DCs for blocking the suppressive effects on exogenous DC in refining DC-based therapy. For example, endogenous immunosuppressive DCs can be transformed to highly immunostimulatory cells to induce robust antitumor responses by the administration of nanoparticles carrying immunostimulatory miRNA miR-155 [12]. In addition, the combination of interleukin-4 (IL-4)/GM-CSF/ tumor lysates (TL)/TNF-*α* induced the greatest differentiation and maturation for DCs in patients with bone and soft tissue tumors in contrast with a combination of IL-4/GM-CSF/TL and a combination of IL-4/GM-CSF/OK-432 [13]. DCs genetically engineered to secrete VEGF (vascular endothelial growth factor) receptor could neutralize soluble VEGF and upregulate expression of costimulatory molecules and proinflammatory cytokines and chemokines, leading to improved antitumor immune response [14]. Similarly, transducing DC with viral vectors that encode immunostimulatory molecules like CD80/CD86 and IL-12 is also a good choice to improved antitumor immunity [15–17]. Furthermore, knock-down of immunosuppressive enzyme indoleamine 2,3-dioxygenase (IDO) in DCs enhanced effective T-cell proliferation and activity and decreased the number of CD4(+) CD25(+) Foxp3(+) Treg cells in a murine breast cancer model [18]. Alternatively, DC-based vaccine in combination with CTLA-4 blockade and depletion of Treg cells via anti-CD25 Ab can improve tumor eradication in a mouse model of colon cancer [19].

## 3. NK Cell-Based Cell Transfer

NK cells, phenotypically defined as CD3^−^CD56^+^ lymphocytes, can rapidly lyse certain target cells without MHC restriction. The NK cell cytotoxicity is mainly dependent on the balance between activating and inhibitory signals [20–22]. The inhibitory receptors of NK cells, including killer cell immunoglobulin-like receptor (KIRs) and CD94/NKG2A/B, can specifically target MHC class I molecules expressed by almost all normal cells and lead to the inhibition of NK cell killing activity [23]. NK cells are activated to kill target cells which have downregulation of MHC-I expression. Therefore, tumor cells that express low MHC-I molecules to evade immunosurveillance are the ideal target cells for NK cells to exert antitumor effect [24]. Certain MHC-I-sufficient tumor cells are also rejected by NK cells that detect stress-induced self-ligands through their activating receptors such as the NKG2D receptor [25]. Therefore, NK cell-mediated cell lysis can be enhanced by using antibodies blocking NK inhibitory receptors or antibodies targeting activating receptors. For example, antibody blocking KIR significantly promoted NK cell Ab-dependent cellular cytotoxicity (ADCC) responses in a human cancer model [26]. Another experiment demonstrated using RNA interference that there was observed silencing NKG2A and increased NK cell lysis by 40% [27]. On the other hand, novel therapies targeting NKG2D ligands expressed on tumor cells rather than normal cells have achieved preclinical success and are currently under investigation in clinical trials [28, 29].

The therapeutic NK cells can be derived from several sources including autologous NK cells, allogeneic NK cells, NK cell lines, genetic modified NK cells, hematopoietic stem cell (HSC), and induced pluripotent stem cells (iPS) [23]. By cytokine stimulation, autologous NK cells can be transformed into lymphokine-activated killer (LAK) cells and exhibit greater cytotoxicity against tumor cells [23, 30, 31]. In 1985, Rosenberg et al. introduced the LAK cells to the treatment for metastatic melanoma refractory to standard therapies. Eleven of 25 patients exhibited objective cancer regression including one experienced complete tumor regression up to 10 months. This exciting achievement ushered in a new era of adoptive immunotherapy. However, a clinical response rate of about 15–20% was reported in all later phase II and phase III trials using LAK therapy in combination with IL-2 therapy. This rate was not superiorer to IL-2 therapy alone [32]. The low frequencies are explained by the prevention of NK cell attack onto “self”-MHC-expressing tumor cells from the inhibitory KIRs or the expansion of regulatory T cells induced by IL-2 [33]. Alloreactive NK cells with KIR-ligand mismatch between patients and their donors can overcome this obstacle and exhibit greater tumor-killing activity in acute myeloid leukemia (AML) without causing graft-versus-host disease [34–36]. As for solid tumors, alloreactive NK cells also play a therapeutic role with minimal toxicity [37]. A phase I clinical trial reported that adoptive transfer of allogeneic NK cells activated and expanded with IL-15 and hydrocortisone (HC) in vitro are safe and potentially effective in combination with chemotherapy in patients with advanced non-small-cell lung cancer [38]. This approach has a disadvantage that KIR mismatched allogeneic NK cells can generate immune-mediated rejection due to MHC mismatch [39].

Compared with autologous or allogeneic NK cells, the NK cell lines expanded under good manufacturing practice (GMP) conditions can supply sufficient quantity for clinical adoptive therapy with easier and simpler procedures. Among NK cell lines, NK-92 cells are the only ones approved by FDA for investigational treatment of patients with advanced malignant melanoma and renal cell carcinoma [40–43]. However, other NK cell lines such as KHYG-1 cells and NKL cells demonstrate a greater antitumor effect than NK-92 cells, holding therapeutic promise [44, 45].

Strong evidence supports that genetic modification of NK cells is another effective strategy to increased tumor-cell killing efficiency. The genetic manipulation approaches include the transgenic cytokine expression, upregulation of activating receptors, silencing inhibitory receptors, and redirecting NK cells via chimeric tumor-antigen specific receptors [23]. Recently, tissue-specific NK cells were found to express divergent phenotypes and play different immunological roles according to the organs [46]. For example, hepatic NK cells can kill tumor cells which are otherwise resistant to splenic NK cells [47]. Therefore, genetically modified tissue-specific NK cells might be used for the treatment of cancers that originated from different organs.

So far, ACT with NK cells has shown promising results mainly in hematological cancer patients [33, 48, 49]. In contrast, NK cell-based therapy in solid tumors proved still unsatisfactory. For example, in a clinical trial of 8 patients with metastatic melanoma or renal cell carcinoma they were treated with autologous NK cells after receiving a lymphodepleting chemotherapy regimen, no tumor regression was observed, although high levels of circulating NK cells persisted for several months [48]. The activation of NK cells prior to transfer into donors seems to be critical. For example, NK cells preactivated with IL-12/IL-15/IL-18 rather than IL-2 proliferated rapidly, produced high level of interferon *γ* (IFN *γ*), and induced pronounced tumor regression [50].

Taken together, NK cell-based therapies have only achieved modest clinical success in cancer patients and more developments are necessary to improve the clinical efficacy of NK cell-based cancer treatment such as the in vitro activation of NK cells.

## 4. T-Cell-Based Cell Transfer

### 4.1. Cytokine-Activated T-Cell

Cytokine-activated T cells are a heterogeneous population of CD3- and CD56-positive, nonmajor histocompatibility complex (MHC)-restricted, natural killer (NK)-like T lymphocytes derived from peripheral blood lymphocytes (PBL), and expanded in vitro by anti-CD3 monoclonal antibody and various cytokines such as IL-2, IL-1, and IFN *γ* [51]. Cytokine-activated T cells have enhanced cytotoxic activity compared to lymphokine-activated killer (LAK) cells. In addition, CIK cells cocultured with DC can generate DC-CIK cells possessing the features of strong proliferation, significant cytotoxic, sufficient secretion of cytokines, and high expression of TCRs. Currently, CIK cells are in testing for applications to a broad spectrum of cancers such as renal carcinoma [52], gastrointestinal cancer [53], advanced NSCLC (non-small-cell lung cancer), [54] and hepatocellular carcinoma [55]. These clinical trials confirmed the safety of ACT with CIK cells but suggested limited efficacy. It is anticipated that the clinical efficacy could be greatly improved by adding antibodies specific to tumor associated antigen (TAA) [51] or engineering CIK cells with tumor-receptor molecules [56–58] or IL-2 gene [59].

Tumor-infiltrating lymphocytes (TILs), isolated form freshly resected tumors, can mediate lysis of tumor cells after in vitro incubation with IL-2. The main effective cells of TILs are CD8+ T cells, whose antitumor function can be boosted by IL-2. Rosenberg and his colleagues treated patients with metastatic melanoma by adoptive transfer of TILs for the first time in 1988, and the efficacy turned out to be not satisfied [60]. Until 2002, exciting results came due to the preparation of a lymphodepleting regimen before adoptive transfer. In this clinical trial, ninety-three patients with measurable metastatic melanoma refractory to standard therapies were infused with autologous TILs in conjunction with IL-2 administration following a lymphodepleting preparative regimen (chemotherapy alone or with 2 or 12 Gy irradiation). The objective response rates, valued by Response Evaluation Criteria in Solid Tumors (RECIST), ranged from 49% to 72%, and the rate increased with greater degree of lymphodepletion. A complete tumor regression was observed in 20 of 93 patients (22%), and this response continued for 37 to 82 months in 19 patients [61]. The elimination of suppressor T cells or reduction of endogenous lymphocytes that can compete for homeostatic regulatory cytokines like IL-7 or IL-15 could be the reason for host lymphodepletion to augment TILs functionality [62, 63]. Despite this encouraging result, ACT with TILs has some obvious disadvantages. First, it is costly and time consuming. Second, CD4+ CD25+ T cells (Treg cells) in TILs can downregulate the antitumor response and IL-2 in culture environment can stimulate the expansion of Treg cells [64]. Third, only about 50% percent of melanomas reproduce antitumor TILs, while many other cancer types rarely contain sufficient tumor-reactive lymphocytes for the identification and expansion of TILs [65–67]. Finally, the lymphodepletion requires better physical condition of patients. New advances in the immunology will provide more practical approaches to improve the clinical efficacy of TILs. For example, the replacement of IL-2 by IL-7 and IL-15 can effectively prevent Treg proliferation [68]. Alternatively, anti-CD25 monoclonal daclizumab can induce long-lasting depletion of CD25(+) Tregs [69, 70].

In addition to cytokines, chemicals in culture medium can also influence the function of T cells after transplantation. For instance, rapamycin-treated T cells acquire an antiapoptotic and rapamycin-resistant phenotype, resulting in better in vivo persistence after transplantation [71].

### 4.2. Genetically Modified T-Cell

To overcome the above-mentioned limitations, another approach was developed based on the genetic modification of autologous lymphocytes from cancer patients. T-cell functions can be significantly improved by genetic modifications such as downregulation of BH3-interacting domain death agonist (BID) [72, 73] or upregulation of BCL-XL and BCL-2 [74, 75], insertion of CTLA4 mutant gene [76], transfection of human c-fms gene for responding to colony-stimulating factor-1 (CSF-1) [77], transduction of C-X-C chemokine receptor type 2 (CXCR2), C-C chemokine receptor type 4 (CCR4) or CCR2B for better migration [78], and modification to express PD1-CD 28 chimaera to overcome immune suppression [79]. In addition, T cells can be equipped with genes that encode receptors capable of recognizing cancer-specific antigen.

There are two types of antitumor receptors, one is the naturally occurring T-cell receptors (TCRs) and the other one is chimeric antigen receptors (CARs). TCR is a heterodimer of *α*- and *β*-chains that recognize antigenic peptides presented by MHC molecules while CAR target antigens in non-MHC-restricted manner through single-chain variable fragments (scFv) [80].

T-cell receptor genes can be derived from effective TILs [65] in melanoma patients or transgenic mice that express human MHC [81]. In addition, bacteriophages can generate tumor-specific TCR genes as well [82]. Then, T cells are transduced with a retrovirus or *Lentivirus* vector containing TCR genes for recognition of TAAs. Morgan and his colleagues achieved the first success by applying T cells transduced with TCR that targeted the MART-1 (melanocyte/melanoma differentiation antigen) to 15 progressive metastatic melanoma patients. Among them, 2 patients showed objective response (1 patient with complete response and 1 patient with partial response) with sustained levels of engineered T cells (between 20% and 70%) at 1 year after infusion [83]. Although the response rate in this small trial appeared lower than that in TIL ACT trial [61], the application of TCR gene-modified T cells proved to be effective and was extended to other solid cancers like synovial cell sarcoma, colorectal cancer, and renal cell carcinoma in clinical trials [67, 81, 84, 85].

The selection of tumor-specific antigens seems to be crucial. A follow-up study with TCRs recognizing MART-1 and gp100h antigens on melanomas reported on-target toxicities such as vitiligo, uveitis, and auditory toxicity because of low-level expression of MART-1 and gp100h on normal melanocytes [67]. In addition, recognition of an unrelated peptide expressed by unrelated protein expressed by cardiac myocytes caused severe cardiovascular toxicity [86]. Therefore, the biggest challenge is to find TAAs not expressed in normal tissues. Cancer testis antigens are promising targets since they are expressed in a variety of epithelial cancers and the testis rather than in normal adult tissues and the absence of class I MHC molecules prevents the testis from T-cell-induced damage. In the clinical trial to evaluate the clinical efficacy of autologous T cells transduced by TCR against NY-ESO-1 (one cancer testis antigen) to treat patients with metastatic synovial cell sarcoma and melanoma, objective partial responses were achieved in 4 of 6 patients with synovial cell sarcoma and 5 of 11 patients with metastatic melanoma. Importantly, no patients exhibited on-target toxicity [86]. In addition to NY-ESO-1, there are more cancer testis antigens under investigation such as MAGE-A3 and SSX2.

However, there are some drawbacks of TCR gene-modified T cells. The *α*- and *β*-chains of introduced TCR can mispair with the corresponding chains of endogenous TCRs, which remarkably reduce the expression of TCRs on the surface of transduced T cells, lending to reduce antitumor efficiency. A creative approach to decrease mispairing is the introduction of transgene encoding small interfering RNA or zinc finger nucleases to downregulate endogenous TCR [87, 88]. Besides, the MHC-restricted nature of TCR function makes it possible that tumor can inhibit T cells recognition of antigens by downregulating MHC expression [89]. For the same reason, TCRs can only response to peptides presented by MHC molecules and fail to be directed to carbohydrate and glycolipid antigens.

In contrast to TCRs, CARs can overcome these barriers. CARs are genetically engineered immunity receptors that bind specific antigens expressed on the surface of cancer cells and then activate T cells to eliminate tumor cells [90]. Generally, CAR consists of an extracellular TAA-specific single-chain antibody variable fragment (scFv), a molecular hinge region, transmembrane domains, and intracellular signaling domains. Single-chain antibody variable fragment can target tumor antigens in non-MHC-restricted manner; therefore the technique can be applied to all individuals irrespective of their HLA type, and it also can recognize carbohydrate and glycolipid antigens [91, 92]. The transmembrane domains participate in dimerization of CARs and activation of T cells. The biggest advantage of CAR over TCR is that CAR can recognize TAAs in a non-MHC-restricted manner. Moreover, NK cells [93] and CIK cells can be modified with CARs as well. The first generation of CAR consists of scFv and ITAM (immunoreceptor tyrosine-based activation motif). Although the feasibility of CARs-expressed T cells was proved by initial experiments [94], transient proliferation of T cells and low-level secretion of cytokines made antitumor activity of T cells unsustained. The second generation of CARs was constructed by the incorporation of co-stimulatory domains such as CD28, CD27, CD134, CD137, CD244, inducible costimulation (ICOS), and leukocyte C-terminal SRC kinase (LCK) into the intracellular signaling domain [95, 96]. Several studies have demonstrated that the second-generation CARs enhanced T-cell function and persistence increased antigen-induced cytokine production and upregulated antiapoptotic proteins, leading to better eradication of established tumors [95, 97–102]. The third-generation CARs have been designed to include additional costimulatory signaling domains (e.g., CD28/4-1BB/CD3*ζ*, CD28/OX-40/CD3*ζ*), which further improve the full signaling capabilities of T cells [103, 104].

The first exciting clinical trial using CARs was carried out by Pule in 2008. They treated 11 neuroblastoma patients with engineered Epstein-Barr virus (EBV)-specific CTLs expressing a chimeric antigen receptor targeted against the disialoganglioside GD2. Tumor necrosis or regressions even sustained complete remission were observed in 4 of 8 patients with evaluable tumors [105]. However, the most impressive and successful achievement was reported in a B-cell lymphoma patient who was treated with T cells expressing CARs directed to the cell-surface protein CD19. CD19 is restricted to normal mature B cells, malignant B cells, B-cell precursors, and plasma cells [106, 107]. The patient experienced a dramatic lymphoma regression [108]. In another encouraging clinical trial, 8 patients with advanced B-cell malignancies were treated with chemotherapy followed by anti-CD19-CAR-transduced T cells and a pulse course of IL-2. Six of the 8 patients obtained objective remissions, while all patients had cells containing the anti-CD19 CAR gene circulating in the blood [109]. Currently, the application of CARs has extended to a variety of cancers like CLL (chronic lymphocytic leukemia) [110], ALL (acute lymphoblastic leukemia) [111], non-Hodgkin's lymphoma [111], acute myeloid leukemia (AML) [112], renal cell carcinoma (RCC) [113], colorectal cancer [94], and ovarian cancer [114].

Unfortunately, there are several side effects to use CAR-engineered T cells. Morgan reported a patient with metastatic colon cancer experienced acute respiratory failure after infusion of anti-ErbB2 chimeric-antigen-receptor-transduced T cells and died five days later. The reason suspected was that modified T cells recognized ERBB2 expressed by normal lung cells and secreted inflammatory cytokines such as TNF-*α* and IFN-*γ*, causing pulmonary toxicity and multiple organ dysfunction syndrome [115].

The differentiation status of T cells is a great concern to maximize antitumor effect of T cells. Gattinoni and his colleagues discovered a human memory T-cell subset with stem cell-like properties, termed memory stem cells (TSCM) which can mediate superior antitumor responses than central memory T cells (TCM) and effector memory T cells (TEM) in a humanized mouse model [116]. The more poorly differentiated T cells possess better proliferative capacity and antitumor effects in vivo [117]. Alternatively, haematopoietic stem cells are also an ideal type to ensure long-term engraftment. Human CD34+ stem cells can be isolated from peripheral blood and genetically modified with tumor-specific receptors. These cells were infused into humanized mice and generated a considerable population of melanoma-specific T cells which can persist long-term in vivo [118].

## 5. Conclusions and Perspectives 

Compared with other standard therapies like chemotherapy, radiotherapy, and surgery, immunotherapy can provide highly targeted treatment owing to the highly specific protein/receptor protein interaction. Activated or engineered immune cells can traffic to cancer tissues to elicit persistent antitumor immune response. Most of current clinical trials confirmed promising albeit moderate clinical efficacy. However, combining immunotherapies or with other traditional therapies such as chemotherapy and radiation has shown potential by preclinical investigations suggesting synergistic effects on tumor response and overall survival [119–122]. In addition, it remains a challenge to define the optimum dose and schedule of combination therapies for best clinical efficacy. The intriguing prospect is that the immunotherapy will occupy an important position and become a standard treatment in cancer.
